# Spectral optimization of supercontinuum shaping using metaheuristic algorithms, a comparative study

**DOI:** 10.1038/s41598-024-84567-x

**Published:** 2025-01-02

**Authors:** Mathilde Hary, Teemu Koivisto, Sara Lukasik, John M. Dudley, Goëry Genty

**Affiliations:** 1 Photonics Laboratory, Tampere University, 33104 Tampere, Finland; 2 Institut FEMTO-ST, Université Bourgogne Franche-Comté CNRS UMR 6174, 25000 Besançon, France; 3 Faculty of Physics, University of Warsaw, Pasteura 5, 02-093 Warsaw, Poland

**Keywords:** Supercontinuum generation, Optics and photonics

## Abstract

Supercontinuum generation in optical fiber involves complex nonlinear dynamics, making optimization challenging, and typically relying on trial-and-error or extensive numerical simulations. Machine learning and metaheuristic algorithms offer more efficient optimization approaches. We report here an experimental study of supercontinuum spectral shaping by tuning the phase of the input pulses, different optimization approaches including a genetic algorithm, particle swarm optimizer, and simulated annealing. We find that the genetic algorithm and particle swarm optimizer are more robust and perform better, with the particle swarm optimizer converging faster. Our study provides valuable insights for the systematic optimization of supercontinuum and other optical sources.

## Introduction

The generation of a broadband supercontinuum (SC) when injecting powerful short laser pulses into a nonlinear optical fiber has revolutionized a wide range of applications in imaging, spectroscopy, and sensing^1^. While the generation of SC spectra spanning very large bandwidth is now routinely achieved in the laboratory, many applications including e.g. spectroscopy or microscopy can benefit from spectral optimization by enhancing the intensity locally in specifically targeted wavelength channels. However, SC generation dynamics are highly nonlinear, and they are difficult to systematically control and optimize in a specific application. This is particularly the case in the anomalous dispersion regime where spectral features arising from soliton fission, dispersive wave generation, and Raman self-frequency shift can yield complex spectral profiles^2^. Often, such spectral optimization requires extensive trial and error iterations, directly in experiments or aided by computationally intensive numerical simulations^3^.

The techniques of machine learning have emerged as a promising tool for smart control of light sources^4–8^, allowing for precise adjustment of input laser parameters to access different operational regimes, and versatile configurations that can support various applications^9^. Among those techniques, the use of metaheuristic algorithms for hardware optimization often proves more efficient than exhaustive mapping of the full parameter space^10^. An optimization problem can typically be reduced to finding the minimum or maximum of a multi-dimensional function, and there are a number of techniques that can used for this. Such techniques can be roughly divided into three categories, depending on their operation principle: (i) classical differential calculus that uses e.g. gradient descent methods^11^; (ii) guided random search techniques based on evolutionary strategies such as a genetic algorithm (GA)^12^, particle swarm optimizer (PSO)^13^, ant colony^14^ or jellyfish optimizer ; (iii) local greedy searches like simulated annealing (SA)^13^. Each algorithm has distinct characteristics and advantages depending on the optimization problem to be solved, and it is generally non trivial to determine the most suitable choice of algorithms for the optimization of a given system^15^.

In the context of SC generation, it was recently shown that a GA can be efficiently used to control the spectral phase of pulses injected into a highly nonlinear fiber for enhancing the spectral intensity in target wavelength channels^16^. Here, we extend this study and compare different optimization algorithms for SC spectral shaping including GA, PSO, and SA. Specifically, we first study the relationship between the topology of an optimization space and optimization complexity using an abstract function commonly used in search algorithm benchmarking^17^. We show that the particular choice of algorithm is not critical for systems with a low number of degrees of freedom, while the GA and PSO are found to perform better for high dimensionality. We then compare the performance of the algorithms in the experimental optimization of SC spectral shaping where a GA, PSO and SA are used to control the spectral phase of femtosecond pulses at 1550 nm injected into a nonlinear fiber. We demonstrate intensity enhancement both in a single and multiple spectral channels in the 1500-2000 nm range, and the performance of each algorithm is evaluated in terms of spectral intensity enhancement and convergence time for different input powers. While we do find some small discrepancy in the performance of the algorithms with the GA and PSO proving to be more robust, the main difference lies in the optimization speed where PSO performs better. Our findings can be of potential interest for the systematic optimization of laser and nonlinear systems in general.

## Results

### Dimensional study

**Fig. 1:**
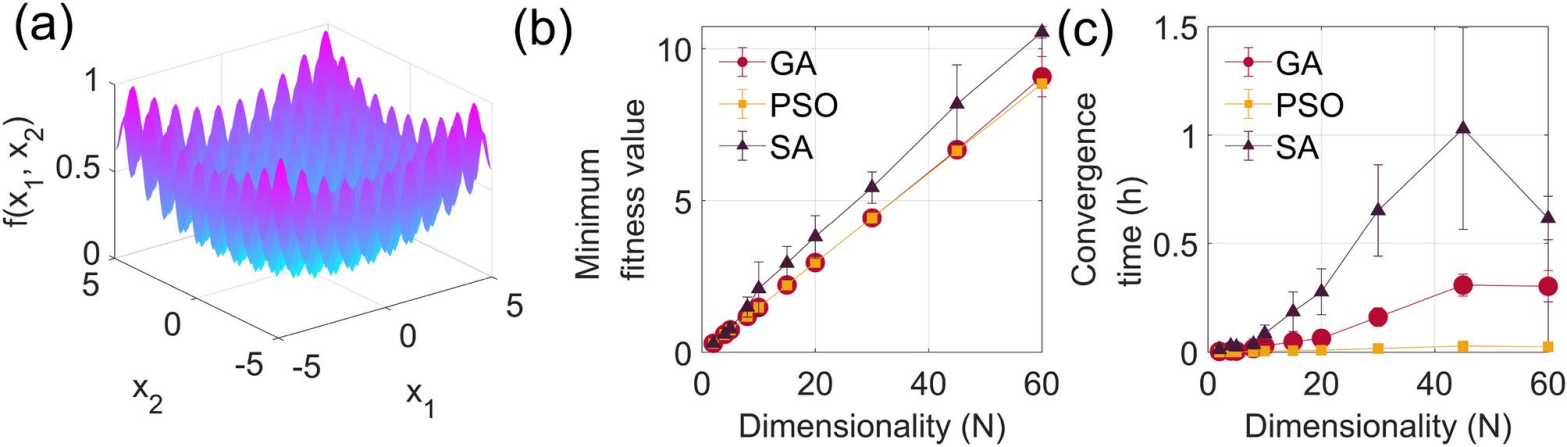
Simulation results of (**a**) the Rastrigin function for N = 2. (**b**) Minimum fitness value and (**c**) time to convergence as a function of Rastrigin function dimensionality for the GA (red dots), PSO (yellow squares) and SA (black triangles). The bars shows the deviation over 10 different runs with different initialization parameters.

We first present results from a general study where we evaluate the performance of different classes of algorithm in the general context of complex system optimization using the Rastrigin function defined as^18^:

1 $$\begin{aligned} f(x_n) = \frac{1}{N}\sum _{n=1}^{N} \left[ \sin (n \cdot x_n) + \cos (n \cdot x_n) + 0.5 \cdot (n \cdot x_n)^2 \right] , \end{aligned}$$

where *N* is an integer $\ge 2$ which determines the function dimensionality. The Rastrigin function is non-convex, with a global minimum surrounded by multiple local minima whose number increases exponentially with the dimensionality. These characteristics make it particularly well-adapted to compare different optimization algorithms and it has been used as a benchmark function to evaluate algorithms optimization performances in different contexts^19–21^. Figure 1a illustrates the Rastrigin function for the particular case $N=2$. The function complexity lies in its topological landscape comporting many local minima. The presence of many local minima can be seen as an “artificial noise” that may be present in a given physical system and the ability of an algorithm to navigate the complex topological landscape of the Rastrigin function and identify the global maximum can provides clear indications on its robustness and performances in a multi-dimensional optimization problem. The Rastrigin function optimization may then provide useful guidance for model-free optimization of nonlinear physical systems, depending on the number of parameters that needs to be optimized.

We apply the GA, PSO and SA (see “Methods”) with the objective of finding the global minimum of the Rastrigin function while varying the number of dimensions *N* from 2 to 60. The results shown in Fig. 1b and c for each algorithm compare the normalized minimum fitness value (corresponding to the global optimum found) and averaged time to convergence evaluated over 10 distinct runs for increasing dimensionality. We use random initialization for each run to (i) reflect the algorithm performance in a real-world scenario where initial conditions may vary and (ii) examine the algorithm’s flexibility and robustness. One can see that for a number of dimensions $2\le N \le 5$ all algorithms essentially show comparable performances, both in terms of minimum fitness value and speed, such that the particular choice of algorithm does not significantly impact the search performance. Above *N* = 10 dimension, however, one observes a more significant discrepancy between the different algorithms. In such high-dimensional scenarios, the PSO and GA can find the global minimum with better accuracy and the PSO converges faster. The SA converges the slowest with increased inaccuracy in finding the global minimum as the dimensionality grows. One may then anticipate that the choice of metaheuristic algorithm for a particular system optimization is not crucial when the system dimension is relatively low. We do note however that SA is more sensitive to the initialization conditions as indicated by the larger deviation in the minimum fitness value. For high dimensional systems however, PSO appears to be more efficient and its ability to navigate complex landscapes in the phase space should have an advantage compared to GA and SA.

### Supercontinuum optimization

We next carried out an experimental comparison of the different algorithms performance for the case of supercontinuum generation to maximize the spectral intensity in specific target wavelength channels. The experimental setup is similar to that described in Ref.^16^. The SC is seeded by 235 fs pulses at 1559 nm produced by a fiber laser injected into a highly nonlinear fiber (HNLF). The SC generation dynamics can be tuned by changing the input spectral phase of the injected pulses via a spatial light modulator (SLM) arranged with a grating and an imaging lens in a 4-f configuration (see “Methods” for details). The applied spectral phase on the SLM is expanded into a fourth-order polynomial $\phi (\omega ) = c_2(\omega -\omega _0)^2+c_3(\omega -\omega _0)^3+c_4(\omega -\omega _0)^4$ where the parameters $c_2$, $c_3$, and $c_4$ are in the range $\pm 15 \times 10^{-27} \mathrm{s^2/m}$, $\pm 15 \times 10^{-41}\mathrm{s^3/m}$, and $\pm 15 \times 10^{-56}\mathrm{s^4/m}$, respectively, and physically correspond to second, third, and fourth dispersion coefficients (note that modifying the spectral phase intrinsically affects the temporal pulse duration). The parameter $c_1=2\pi c/\omega _0$ (*c* is the speed of light in vacuum) in the range 1540-1570 nm determines the minimum of the input spectral phase which may be offset from the laser center wavelength. We have checked that including additional dispersion terms beyond fourth order have a negligible effect on the SC dynamics. Finally, an additional control parameter $c_5$ represents the average power of the injected pulses train that can be tuned with the SLM by 30 % from a maximum nominal value set by the user, corresponds to a range where the supercontinuum experiences significant spectral narrowing, when combined with the other parameters. The search space therefore includes five different parameters corresponding to a dimension $N=5$. Furthermore, in our supercontinuum system, we identify two different types of potential noise sources with different time scales. The first type corresponds to shot-to-shot fluctuations of the injected laser pulses (note that we are operating in the coherent regime of SC generation and therefore the fiber nonlinearity does not amplify these fluctuations). This type of noise can be neglected since the SC spectrum is measured with an optical spectrum analyzer that averages over many consecutive pulses. The second source of noise corresponds to slow drifts in the experiment induced by temperature or humidity variations in the environment. Here, the typical convergence time of the optimization algorithms is significantly shorter ( 20 min) than potential experimental drifts (typically some hours) which minimizes the impact on the optimization.

### Single spectral channel optimization

**Fig. 2:**
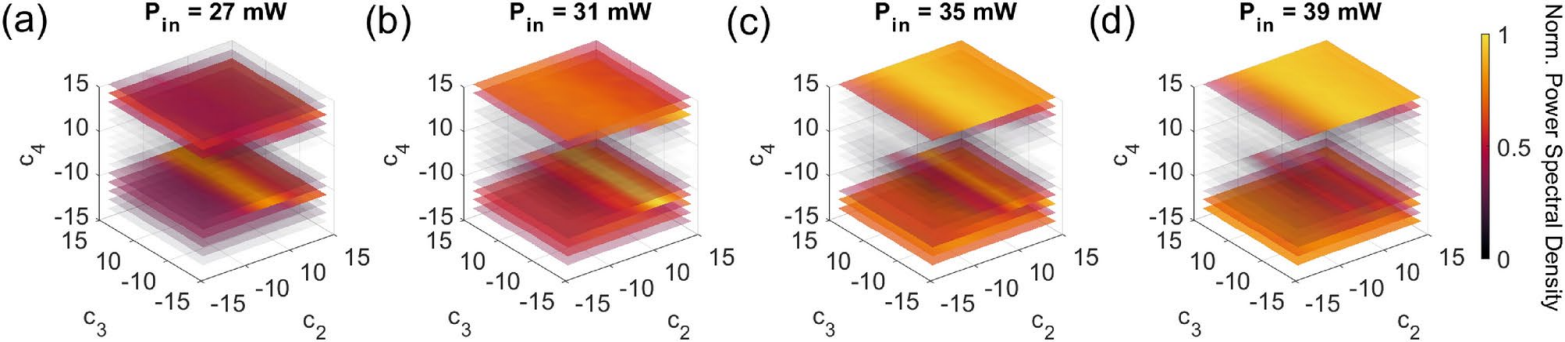
Experimental results of the linear grid search for the supercontinuum system of different fixed injection power: (**a**) 27 mW, (**b**) 31 mW, (**c**) 35 mW and (**d**) 39 mW, for $\lambda _{T}$ = 1600 nm.

For reference, we first characterize the tuning parameter space by mapping the optimization space through an exhaustive grid search for optimizing the power spectral density inside a 7 nm bandwidth channel centered at the target wavelength $\lambda _{\text {T}}$ = 1600 nm. We choose this bandwidth as it corresponds to the typical spectral feature size of the generated supercontinuum. Figure 2 shows the result of the systematic grid search as a false color plot at several fixed injected powers values $P_{\text {in}}$ of 27 mW, 30 mW, 35 mW and 39 mW while systematically scanning the parameters $c_2$, $c_3$, and $c_4$. Note that we restrict the systematic grid search to three of the five dimensions of optimization space (the second, third and fourth order dispersion terms) as incorporating more parameters would exponentially extend the scanning time. The sub-plots are normalized to the maximum integrated power value around 1600 nm in a 7 nm bandwidth and the lowest values were made transparent to highlight the most relevant features of the optimization space. The grid search reveals multiple possible solutions and a smooth landscape with low complexity. We performed additional grid-search for different spectral channels at 1700 nm, 1800 nm, and 1900 nm, respectively, and we observed similar characteristics. The exhaustive grid search takes 100 minutes, which is too slow for practical application purposes. We then apply the GA, PSO and SA algorithms to search for the optimal tuning parameters to optimize the SC power spectral density in a target spectral band.

Compared to our previous work reported in Ref.^16^ where the fitness function is defined at the energy in a spectral window, we use here a Gaussian-shaped filter centered at $\lambda _{\text {T}}$ and spectral width $\sigma$ to define the fitness function:

2 $$\begin{aligned} \text {F}(\lambda _{\text {T}} ) = \int _{\Delta \lambda _{\text {T}}}\text {S}(\lambda ) \times \exp \left[ -\frac{(\lambda - \lambda _{\text {T}})^2}{2\sigma ^2}\right] \mathrm d\lambda , \end{aligned}$$

where $\text {S}(\lambda )$ represents the SC spectrum. This particular form of the fitness function ensures that a large fraction of the optimized intensity is focused near the center of the spectral channel. Specifically, compared to the fitness function used in^16^ which computes the integrated the power over the target channel bandwidth, the addition of the Gaussian filter improves the performance in about 60 $\%$ of the tested 50 channels. We also extend the boundaries of the tuning phase parameters as the linear scan showed the best solution to be found at the edge of the search range in the high power regime. The search intervals are now defined as $c_2 \in \pm 9\cdot 10^{-26}~\mathrm s^2/m$, $c_3 \in \pm 9\cdot 10^{-40}~\mathrm s^3/m$, $c_4 \in \pm 9\cdot 10^{-55}~\mathrm s^4/m$. To quantify the optimization, we define the enhancement factor $\eta (\lambda _{\text {T}})$ for a given target spectral channel at $\lambda _{\text {T}}$ as the ratio between the optimized power $P_{\text {final}}$ to the initial power $P_{\text {initial}}$ integrated over a given spectral bandwidth $\Delta \lambda _{\text {T}}$:

3 $$\begin{aligned} \eta {(\lambda _{\text {T}})} = \frac{\int _{\Delta \lambda _{\text {T}}} P_{\text {final}}(\lambda _{\text {T}}) \, \mathrm d\lambda }{\int _{\Delta \lambda _{\text {T}}} P_{\text {initial}}(\lambda _{\text {T}}) \, \mathrm d\lambda }. \end{aligned}$$

**Fig. 3:**
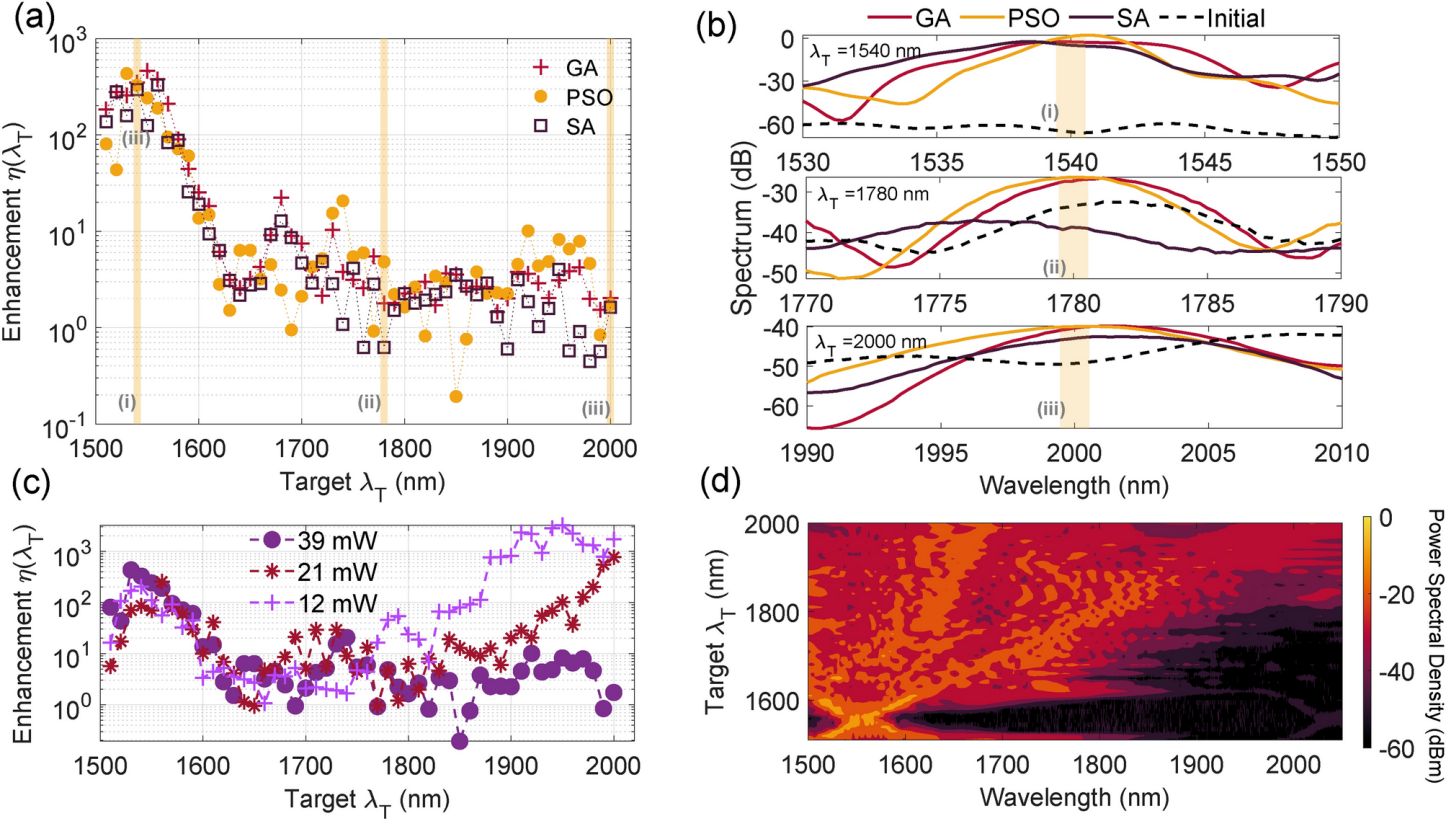
Experimental results of (**a**) the enhancement factor $\eta (\lambda _{\text {T}})$ as a function of target spectral channel for the three different algorithms. (**b**) Detailed view of optimized spectrum around three distinct target spectral channels whose location is marked by the shaded rectangular area. (**c**) Enhancement obtained by PSO for increasing input powers. (**d**) SC spectrum at the fiber output when the optimized target spectral channel (using PSO) is varied from 1500 to 2000 nm at an injected power $P_{\text {in}}$ = 21 mW.

Figure 3a compares the enhancement factor obtained by the different algorithms for a single target spectral channel $\lambda _{\text {T}}$ from 1500 to 2000 nm with bandwidth $\Delta \lambda _{\text {T}}$ = 7 nm and at a maximum injected average power of $P_{\text {in}}=39~$ mW. One can see that, for channels with wavelength near that of the pump laser, the enhancement factor is in the range $\eta$ = 100-400 and decreases to $\eta$ = 2-10 for channels in the long wavelength edge. Those results also show that in general, the enhancement factor is of the same order of magnitude for all algorithms. To better illustrate the similarity in performance of the different algorithms, we plot in Fig. 3b the optimized spectrum at three distinct spectral channels close to the pump laser wavelength with $\lambda _{\text {T}}$ = 1540 nm, at an intermediate detuning with $\lambda _{\text {T}}$ = 1780 nm, and near the SC long wavelength edge $\lambda _{\text {T}}$ = 2000 nm (corresponding to the shaded rectangular areas in Fig. 3a). The enhancement factors for the GA, PSO and SA are $\eta _\text {PSO}$ = 327/4.8/1.7, $\eta _\text {GA}$ = 348/1.8/2 and $\eta _\text {SA}$ = 293/0.6/1.6 at $\lambda _{\text {T}}$ = 1540/1780/2000 nm, respectively. This is not particularly surprising, in agreement with the analysis based on the Rastrigin function since the dimensionality of the optimization problem here is rather low with $N=5$. Note that for a few specific channels $\eta (\lambda _T) < 1$, which means that the algorithms are not able to converge towards an optimized solution. This is attributed to experimental fluctuations in the laser power and drift in input coupling over time.

In an attempt to answer the question whether one algorithm performs better overall, we computed a mean enhancement factor for target channels in the range 1500-2000 nm normalized to the best enhancement value among the three algorithms. In other words, if this average normalized factor for a particular algorithm is equal to one, it would indicate that the algorithm always yield better enhancement that the others. We found that PSO and GA exhibits similar performance with a mean normalized enhancement of 0.75 and 0.79, respectively while the SA showed the least performance on average with a mean normalized enhancement of 0.58. This difference in the SA performance can be explained in the light of the dimensional study performed above on the Rastigin function which shows that SA is more prone to deviations in optimization as compared to GA and PSO for a random set of initial conditions. Although the PSO and GA algorithms have similar enhancement performance, it is worth emphasizing that the PSO converges faster: 17 minutes compared to 20 minutes for the GA and SA on average.

We next focus on the performance of the PSO due to its faster convergence. Specifically, we repeated the single channel optimization at different maximum injected power. The results are shown in Fig. 3c for maximum injected power values of $P_{\text {in}} = 12, ~21, \mathrm and ~39 ~$mW. Enhancement is achieved independently of the power level, confirming the robustness and efficiency of the PSO in achieving consistent optimization. We also note the larger enhancement factor in the long wavelength side of the SC spectrum at lower injected power. This is because when no spectral phase is applied the SC broadening is reduced up to 1900 nm and 1800 nm for $P_{\text {in}}$ = 21 mW and 12 mW, respectively, yielding very large enhancement when the SC spectrum extends further by applying a particular phase profile.

Figure 3d shows as false color plot the optimized SC spectrum as a function of the optimized spectral channel $\lambda _{\text {T}}$ for $P_{\text {in}}$ = 21 mW. Each spectrum was normalized to the maximum spectral intensity value from all optimized spectra. The figure highlights the enhancement in the spectrum when a particular wavelength is targeted with a distinctive feature on the diagonal at $\lambda$ = $\lambda _{\text {T}}$. We can also see that optimization generally leads to larger power value for target channels around the pump laser wavelength. For channels with wavelength below 1800 nm, we observe a sharp spectral focusing from either side of the pump (appearing as a cross centered around the pump wavelength)rather than a fully developed SC. For channels at longer wavelengths, the optimized SC spectrum is generally much broader, resulting in reduced power in the optimized channel.

### Multi-wavelength optimization

**Fig. 4:**
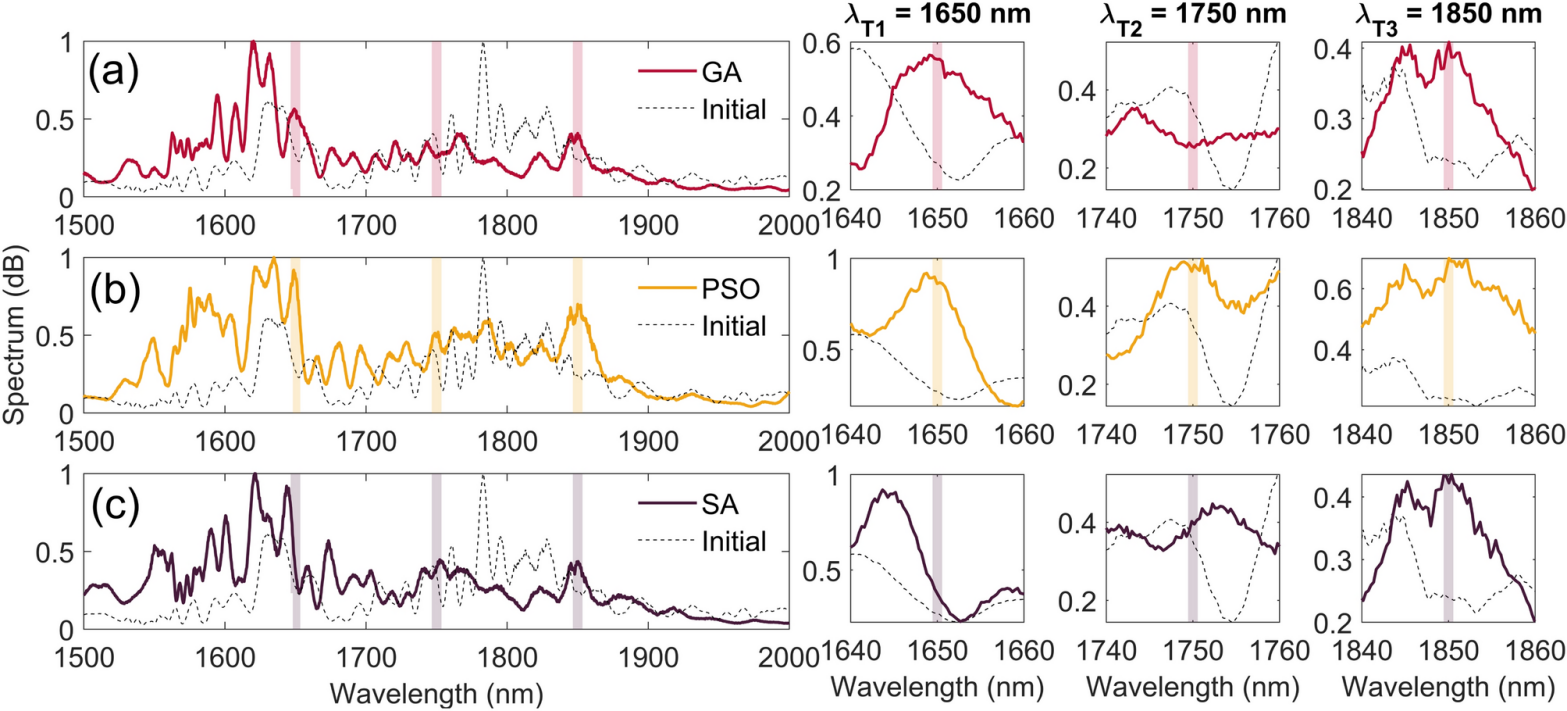
Multi-wavelength optimization with zoomed spectrum around the target channels at $\lambda _{\text {T1}}$, $\lambda _{\text {T2}}$, $\lambda _{\text {T3}}$ for (**a**) GA, (**b**) PSO, and (**c**) SA. Dashed line represent the initial spectrum, without any shaping, solid line are spectrum after optimization. Inset on the right are zoomed regions around optimization targets.

We also compared the performance of the different algorithm for simultaneously optimizing the spectral power in three different spectral channels. The fitness function used in this case is defined as:

4 $$\begin{aligned} \text {F}(\lambda _{\text {i,j}} ) = -\prod _{i=1}^{3} \prod _{j=1}^{3} \int _{\Delta \lambda _j}\text {P}(\lambda -\lambda _i)\mathrm d\lambda , \end{aligned}$$

where P represents the power around a target channel centered at $\lambda _i$ and integrated over a spectral bandwidth $\Delta \lambda _j$. Note that similarly to Ref.^16^, we use here the product of the integrated power in the individual channels rather than the summation to avoid enhancement in one particular spectral channel at the expense of the others. We also perform integration over three different wavelengths intervals ($\Delta \lambda _j$ = 1 nm, 5 nm and 10 nm for i=1, i=2 and j=3, respectively) to concentrate the power around the center of the spectral channel. Figure 4 illustrates an example of results obtained for target channels at $\lambda _{\text {T1}}=1650$ nm, $\lambda _{\text {T2}}=1750$ nm, and $\lambda _{\text {T3}} =1850$ nm, where one can see that all three algorithms -(a) GA, (b) PSO, and (c) SA- effectively enhance the power in the three spectral channels simultaneously, except at $\lambda _{\text {T2}}$ in the case of the GA. To better highlight the difference in performance, we further show in the figure a detailed view of the SC spectrum around the target spectral channels. This detailed view reveals that PSO performs slightly better than the other algorithms, and this was also observed for other combinations of spectral channels. However, it is also important to emphasize that it was not always possible to achieved simultaneous enhancement for any arbitrary combination of spectral channels and, this, regardless of the algorithm used. This highlights that multi-channels optimization is highly dependent on the specific characteristics of the SC which are determined by the nonlinear fiber parameters and fixed in the experiment.

## Conclusion

**Fig. 5:**
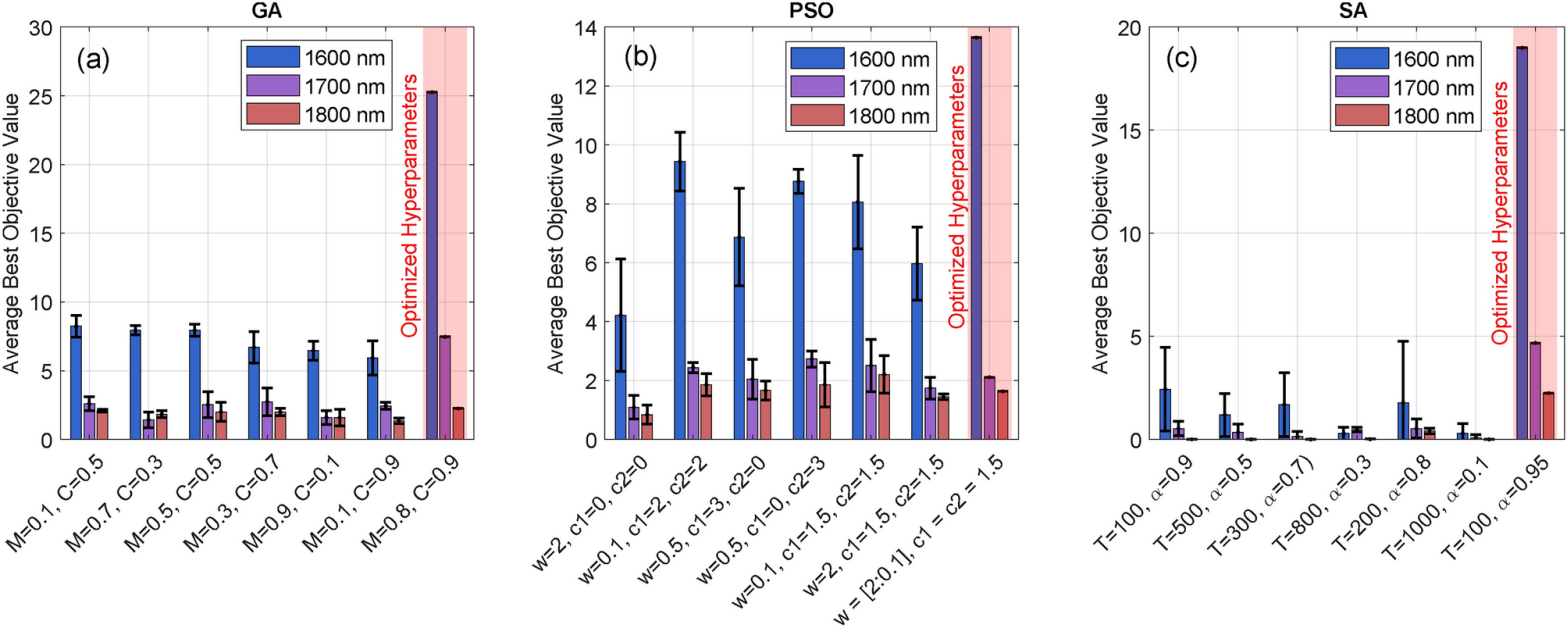
The figure presents the average best objective values obtained using different configurations of main parameters for (**a**) GA, (**b**) PSO, and (**c**) SA. The results demonstrate the impact of parameter tuning on optimization performance across wavelengths (1600 nm, 1700 nm, and 1800 nm).

We have studied the optimization of supercontinuum generation in a highly nonlinear fibers using different metaheurstic algorithms. The insight derived from this comparative study where the algorithms are applied to enhance spectral intensity in targeted spectral bands is that the particular choice of algorithm is not critical when the number of optimization parameters is relatively low. For systems with a relatively number of optimization parameters, the choice of algorithm generally does not significantly impact the performance. For single-channel optimization, enhancement factors reaching up to few hundred are obtained for all the algorithms, depending on the spectral channel and injected power. For multi-wavelength optimization, the particle swarm optimizer provides slightly better performance, and it was also found to exhibit faster convergence speed. Our results can potentially contribute to the development of more efficient and tunable SC systems. Finally, while here we have focused on comparing the optimization performance of GA, PSO, and SA, it is important to acknowledge that there are many more other algorithms that could potentially offer improved performance or better suitability for specific applications. In particular for the multi-channels optimization, here we use a single-objective cost function but approaches based on multi-objective functions and pareto front optimization which could be more efficient. Exploring these additional algorithms or approaches could further contribute to develop more systematic optimization strategies of supercontinuum sources (Fig. 5).

## Methods

### Experimental setup

**Fig. 6:**
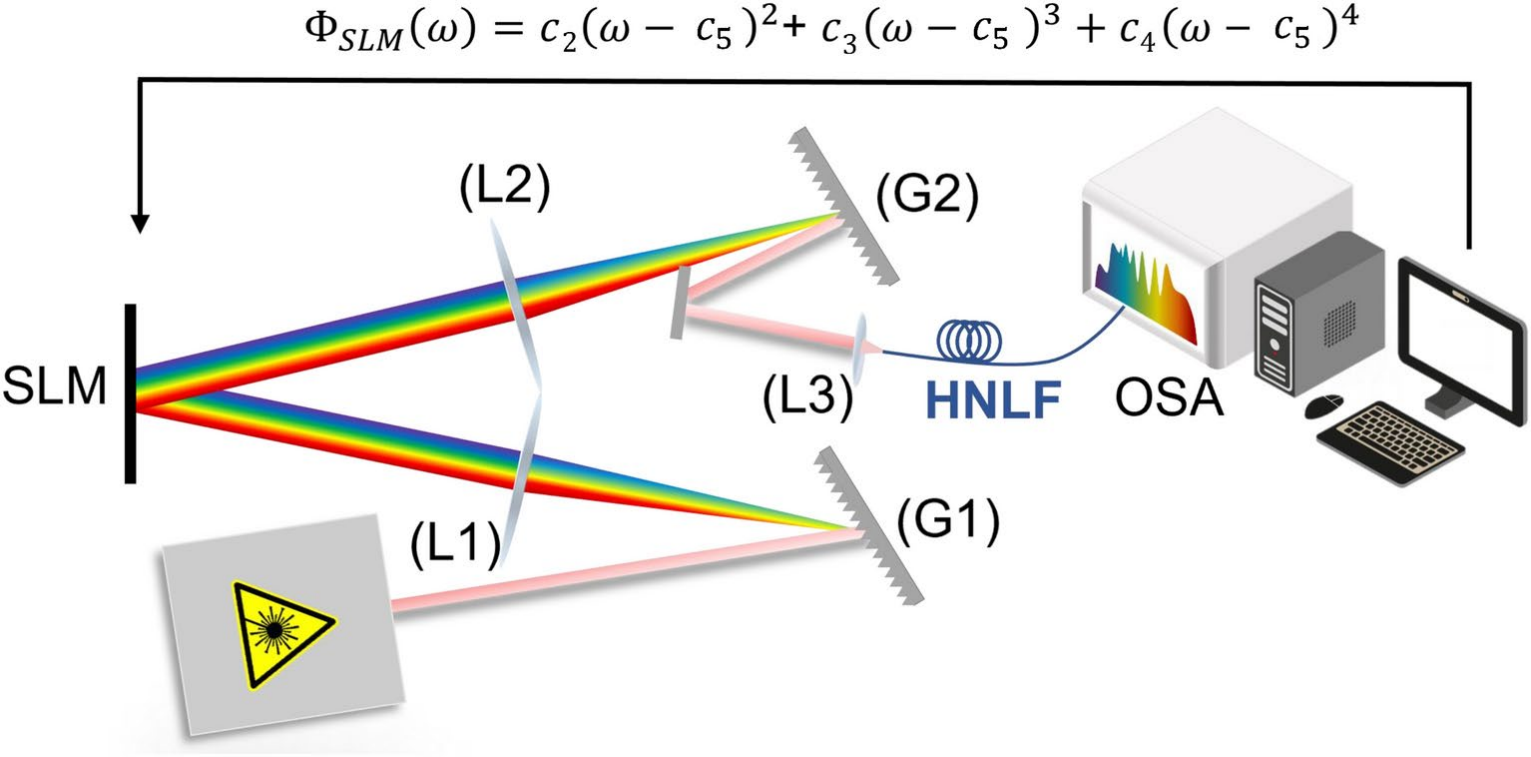
Experimental setup for spectrum optimization adapted from Ref.^16^. (G): Grating, (L): Lens, SLM: Spatial Light Modulator, HNLF: Highly Nonlinear Fiber, OSA: Optical Spectrum Analyzer. $\Phi _{SLM}{(\omega )}$: phase pattern, $c_{2}$, $c_{3}$, and $c_{4}$ are the input parameters for the optimization algorithms, respectively, quadratic, cubic, quartic phase coefficient and $c_{5}$ the central frequency of the phase pattern.

The experimental setup used in this study is based on a previously described configuration^16^ and is shown in Fig. 6. We use a 4-f line with a SLM (Holoeye Pluto 2.1) placed in the Fourier plane in a folded configuration. The pump laser pulse duration before entering the fiber is 235 fs, at a repetition rate of 40.9 MHz with 14 nm FWHM, and the beam diameter is 5 mm. The fiber used in this study exhibits a dispersion close to zero at the pump wavelength, *D* = -0.1 $\mathrm ps/(nm^2.km)$ at 1550 nm. The fiber is 5 meters long with 20 cm single-mode patch cord on each side. The zero-dispersion wavelength is located at 1555.9 nm and the fiber mode field diameter is 3.85 $\upmu$m. For an input power of 39 mW, the SC spans over an octave from 1200 nm to 2200 but we focus on the 1500-2200 nm band to match the optical spectrum analyzer (Yokogawa AQ6376) measurement range. The SLM is updated in a loop with different input parameters. The coding was conducted in MATLAB utilizing the Global Optimization Toolbox.

### Algorithms parameters

We employed three different optimization algorithms, each with different operation principles. All algorithms were implemented using the MATLAB Global Optimization toolbox and were adapted to ensure the same number of measurements, the same tolerance values, and the same number of stall generations (i.e. number of generations in a row with unchanged fitness value in a row). In order to optimize the hyperparameters of the different algorithms, seven different configuration were experimentally tested for three different target channels (1600,1700 and 1800 nm), and averaged over three runs with distinct initialization conditions. Figure 5 shows the performance comparison for the different algorithm and hyperparameters configurations. The GA relies of two main parameters that influences the algorithm convergence. Specifically, mutation *M* allows to maintain diversity and exploration by randomly changing some individuals in a given population. Crossover *C* combines genetic information from parent solutions to generate potential better solutions and is used to refine the better solutions. Generally, we found that a high mutation rate to prioritize exploration over exploitation was beneficial. We also found that using a high crossover fraction to preserve the best solutions and achieve an efficient search was required. For PSO, the main hyperparameters are the cognitive coefficient $c_1$, which steers individual exploration towards the best positions, the social coefficient $c_2$, which promotes collective exploration towards the swarm global optimum, and the inertia weight *w* controlling the influence of the particle previous velocity on its current velocity. A small inertia weight reinforces refining known good solutions, while a larger inertia weight favors exploration by searching new areas of the solution space. In general, a balanced rate of cognitive and social coefficients with a small inertia weight, provide a good starting point for balancing exploration vs. exploitation. SA uses three parameters: the starting temperature *T*, cooling rate $\alpha$, and final temperature $T_{final}$. A high initial temperature allows exploration of diverse regions, and the cooling rate ensures gradual temperature reduction, favoring exploration. A particular feature of SA is that it can accept worse solutions in order to converge to a better one later on. To this end, a high initial temperature enables acceptance of worse solutions, and it should be high enough to explore various regions of the solutions space. The cooling rate defines the rate at which the initial temperature is reduced. High cooling rate reduces the temperature gradually, allowing more exploration, while lower cooling rate allows for faster convergence. Finally the starting point plays a crucial role for the SA. The characteristics and optimized hyperparameters used in our experimental optimization are summarized in Table 1.

**Table 1:** Comparison of optimization algorithms.

	Genetic algorithm (GA)	Particle Swarm Optimization (PSO)	Simulated annealing (SA)
Optimization approach	Evolutionary	Swarm intelligence	Stochastic local search
Population based	Yes	Yes	No
Inspiration source	Genetics and natural selection	Flocking behavior of birds/fish	Annealing process
Main parameters	Population (25), generation (75), mutation (M = 0.8), crossover (C = 0.9)	Swarm size (25), social and weights velocities (c1= 1.5, c2 = 1.5) (w = [2:0,1])	Temperature (T = 100), cooling parameter ( $\alpha$= 0.95)

## Data Availability

The data can be obtained from the corresponding author upon reasonable request. All codes, and Figs. 1, 2, 3, 4, 5, used in the manuscript was generated using the standard Genetic Algorithm (GA), Particle Swarm Optimization, (PSO) and Simulated Annealing (SA), suite within MATLAB’s Global Optimization toolbox.

## References

[1] Alfano, R. R. *The Supercontinuum Laser Source: The Ultimate White Light* (Springer, 2006).

[2] Dudley, J. M. & Taylor, J. R. *Supercontinuum Generation in Optical Fibers* (Cambridge University Press, 2010).

[3] Agrawal, G. P. *Nonlinear Fiber Optics* (Academic Press, 2013).

[4] Brunton, S. L. & Kutz, J. N. *Data-Driven Science and Engineering: Machine Learning, Dynamical Systems, and Control* (Cambridge University Press, 2019).

[5] Strogatz, S. H. *Nonlinear Dynamics and Chaos: With Applications to Physics, Biology, Chemistry, and Engineering* (CRC Press, 2018).

[6] Kutz, J. N., Fu, X. & Brunton, S. Self-tuning fiber lasers: machine learning applied to optical systems. In *Advanced Photonics*, NTu4A.7, 10.1364/NP.2014.NTu4A.7 (Optica Publishing Group, 2014).

[7] Tada, J. et al. Adaptively controlled supercontinuum pulse from a microstructure fiber for two-photon excited fluorescence microscopy. *Appl. Opt.* **46**, 3023–3030 (2007). 17514253 10.1364/ao.46.003023

[8] Lorenc, D., Velic, D., Markevitch, A. N. & Levis, R. J. Adaptive femtosecond pulse shaping to control supercontinuum generation in a microstructure fiber. *Opt. Commun.* **276**, 288–292 (2007).

[9] Boyd, R. W. *Nonlinear Optics* (Academic Press, 2008).

[10] Michalewicz, Z. & Fogel, D. B. *How to Solve It: Modern Heuristics* (Springer, 2004).

[11] Ruder, S. An overview of gradient descent optimization algorithms. *ArXiv***abs/1609.04747** (2016).

[12] Haupt, R. L. & Haupt, S. E. *Practical Genetic Algorithms* (Wiley, 1998).

[13] Kennedy, J. & Eberhart, R. Particle swarm optimization. In *Proceedings of ICNN’95 - International Conference on Neural Networks*, 1942–1948 (1995).

[14] Dorigo, M. & Stützle, T. *Ant Colony Optimization* (MIT Press, 2004).

[15] Holland, J. H. *Adaptation in Natural and Artificial Systems* (MIT Press, 1992).

[16] Hary, M. et al. Tailored supercontinuum generation using genetic algorithm optimized Fourier domain pulse shaping. *Opt. Lett.* **48**, 4512–4515. 10.1364/OL.492064 (2023). 37656541 10.1364/OL.492064

[17] Brown, T. G. & Taylor, H. F. Modal analysis of optical fiber polarization rotators. *Opt. Lett.* **12**, 743–745 (1987).

[18] Rastrigin, L. A. *Systems of Extremal Control* (Mir, 1974).

[19] Saez, Y., Isasi, P. & Segovia, J. Interactive evolutionary computation algorithms applied to solve rastrigin test functions. In *Soft Computing as Transdisciplinary Science and Technology: Proceedings of the fourth IEEE International Workshop WSTST’05*, 682–691 (Springer, 2005).

[20] Yumin, Y., Bolin, L. & Shuai, L. A new optimization algorithm and its comparison on traditional optimization algorithms. In *2019 Chinese Control Conference (CCC)*, 2698–2701 (IEEE, 2019).

[21] Saraswat, M. & Sharma, A. K. Genetic algorithm for optimization using matlab. *Int. J. Adv. Res. Comput. Sci.* **4**, 155–159 (2013).

